# Informal politics and inequity of access to health care in Lebanon

**DOI:** 10.1186/1475-9276-11-23

**Published:** 2012-05-09

**Authors:** Bradley Chen, Melani Cammett

**Affiliations:** 1 Program in Health Care Financing, Harvard School of Public Health, 124 Mount Auburn Street, Suite 410, Cambridge, MA, 02138, USA; 2 Department of Political Science, Brown University, 36 Prospect Street, Box 1844, Providence, RI, 02912, USA

**Keywords:** Informal political institutions, Health inequity, Mixed methods research, Lebanon

## Abstract

**Introduction:**

Despite the importance of political institutions in shaping the social environment, the causal impact of politics on health care access and inequalities has been understudied. Even when considered, research tends to focus on the effects of formal macro-political institutions such as the welfare state. We investigate how micro-politics and informal institutions affect access to care.

**Methods:**

This study uses a mixed-methods approach, combining findings from a household survey (n = 1789) and qualitative interviews (n = 310) in Lebanon. Multivariate logistic regression was employed in the analysis of the survey to examine the effect of political activism on access to health care while controlling for age, sex, socioeconomic status, religious commitment and piety.

**Results:**

We note a significantly positive association between political activism and the probability of receiving health aid (*p < .001*), with an OR of 4.0 when comparing individuals with the highest political activity to those least active in our sample. Interviews with key informants also reveal that, although a form of “universal coverage” exists in Lebanon whereby any citizen is eligible for coverage of hospitalization fees and treatments, in practice, access to health services is used by political parties and politicians as a deliberate strategy to gain and reward political support from individuals and their families.

**Conclusions:**

Individuals with higher political activism have better access to health services than others. Informal, micro-level political institutions can have an important impact on health care access and utilization, with potentially detrimental effects on the least politically connected. A truly universal health care system that provides access based on medical need rather than political affiliation is needed to help to alleviate growing health disparities in the Lebanese population.

## Introduction

Despite the importance of political institutions in shaping the social environment, the causal impact of politics on health and its inequities has been understudied [1,2]. Even when considered, the political system is generally not credited with a direct impact on health care access or is seen to have an indirect influence on utilization via its effect on economic conditions [3]. Furthermore, studies tend to focus on formal political institutions at the national level.

This article argues that political institutions can have an important impact at the *individual* level through informal practices. Focusing on Lebanon, we use a mixed-methods approach to show that political organizations use access to health care as a strategy to gain and reward support, leading to potentially detrimental effects on the most vulnerable and exacerbating health disparities.

## Political institutions and health inequities

Although health is an important aspect of social policy and is highly valued by people around the world, there is only a limited literature on the relationship between politics and population health [1]. Most studies come from the social science literature on “welfare states,” which tends to focus on advanced, industrialized countries [4,5]. In general, studies in this vein contend that political institutions affect population health and health inequities only through shaping welfare policies and determining the resources devoted to social services [6-9]. Nevertheless, the literature is far from reaching a definitive conclusion [10,11].

We argue that these macro-political mechanisms are not the only ways in which politics shape access to health and health outcomes. In this paper, we highlight the role of informal politics in influencing access to health care at the individual level. As we illustrate with the example of Lebanon, informal practices, whereby access to social services, including health, is used as a deliberate strategy by politicians and political parties to gain and reward support, can affect access to health care and exacerbate health inequalities in the population. Our study contributes to emerging research on the dynamics of welfare regimes in the Global South, where public welfare functions are less developed and private actors may play a larger role than in industrialized countries [12-16]. In this context, informal politics can assume a critical role in shaping access to health and health outcomes [17,18].

Lebanon provides an appropriate case study for exploring the relationship between informal politics and access to health care. Based on a power-sharing system, political posts at all levels of government are distributed according to sectarian affiliation while relations between citizens and their elected representatives are characterized by informal, clientelist exchanges. The state has low administrative capacity and patronage and corruption are endemic in the system [19,20]. In the context of weak state institutions, the public sector plays a minimal role in assuring social protection while non-state actors predominate in the welfare regime. A range of non-state providers, including political parties, religious charities, community-based groups, non-governmental organizations, and for-profit institutions, supply and finance health care. Thus, the health sector is an appropriate arena for examining the informal political dynamics of gaining access to social assistance [21]. The next section provides a brief overview of the Lebanese health system as background for the empirical analysis.

## The Lebanese health system

The Lebanese health system is characterized by minimal state regulation and heavy reliance on private, non-state providers. The limited public welfare role in part derives from the historical experience of social organization along sectarian lines [22]. Civil war from 1975 to 1990 consolidated the provision of welfare by sectarian groups as protracted conflict undercut the limited public health infrastructure constructed in prior decades and permitted the unrestricted growth of private health services. Between 1975 and 1991, the share of public hospital beds dropped from 26 percent to 10 percent of the total [23], and more than 50 percent of private hospital bed capacity was concentrated in high-cost curative care. After the war ended, the public sector continued to play a minimal role in the direct provision of health services.

Several political parties and movements operate independent health networks, with their own hospitals, clinics and dispensaries as well as computerized systems for maintaining medical records. For example, various agencies linked to the Shi’i Muslim Hezbollah operate four hospitals and at least 24 clinics while the Shi’i Amal Movement administers about 13 clinics and exerts de facto control over at least two public hospitals. The Sunni Muslim Future Movement effectively manages one public hospital and runs over 40 clinics through the affiliated Hariri Foundation Health Directorate [24]. Yet the weight of political organizations in health care far exceeds services provided in their own facilities: Because political parties use their connections to obtain subsidies provided by the government or these institutions themselves for supporters in private and non-governmental institutions run by co-religionist charities or sympathetic physicians, their influence is broader than the number of health facilities that they administer directly. NGO-run health clinics and dispensaries, including those run by political parties, are critical for lower-income families in urban areas and may be the only facilities available in rural areas [25,26]. Furthermore, high level appointments in the Ministry of Public Health also follow a political logic. For example, the Director General is usually Druze while the Minister may rotate between sects. It is generally accepted in Lebanon that differential access to these officials influences access to coverage for inpatient care.

In contrast to its minimal role in service provision, the government is a major source of health care financing, particularly for secondary and tertiary care. In 2006, the public sector accounted for almost 47 percent of total health expenditures while out-of-pocket expenditures amounted to about 39 percent of health spending. Employers and donors supplied about 10 percent and 2 percent, respectively, of spending on health.

The vast majority of government spending on health goes to the public hospitalization program, which covered 100 percent of hospitalization fees until 1992 and 85 percent afterwards [27], as well as 60 percent of high-tech ambulatory services such as MRIs and CT scanners and 100 percent of cancer drugs [25]. On paper, any citizen is eligible for these benefits. In practice, the program offers opportunities for personal discretion by ministry employees and enables political parties and religious authorities to obtain favors for supporters and congregationists while claiming credit for these benefits. Moreover, because the government hospitalization program is in arrears, some hospitals do not accept patients without demonstrated financial means or without vouchers from powerful political intermediaries. These facts all suggest that political factors shape financial access to health care in Lebanon, particularly for lower-income and needy individuals. The next section presents findings from a household survey that indicates that political activism does play an important role and therefore support our hypothesis.

## Data and methods

Between 2006 and 2009, Cammett carried out field research in Lebanon on the provision of social welfare by political and religious organizations, including in-depth interviews with officials in the Ministries of Public Health and Social Affairs, NGO representatives, officials from political parties and religious institutions, and local journalists and researchers (n = 175). At the same time, she worked with a team of Lebanese graduate students to carry out in-depth interviews with Lebanese citizens from diverse religious affiliations to gather information on their experiences in accessing health care and other forms of social assistance (n = 135). The interviewees were selected through purposive, non-random procedures designed to maximize variation according to religious identity, place of residence, and socioeconomic status.

These interviews provided critical background information for the construction, execution and interpretation of a national household survey, which was conducted in spring 2008 to collect information on access to primary and secondary healthcare, financial aid for health and other forms of social assistance, religious identity and observance, political preferences and participation, and standard demographic questions. The sample was designed as a cross-section of all citizens above the age of 18 in Lebanon. Based on a stratified**,** multi-stage, area probability sample, households were selected randomly based on probability proportionate to population size (PPPS). The sample was stratified by province, reducing the likelihood that distinctive regions, which tend to have varied concentrations of religious or ethnic groups, were left out of the sample. Next, a PPPS procedure was used to randomly select primary sampling units (PSU) in order to guarantee that more populated geographical units had a greater probability of being chosen. Households were then randomly selected within each PSU. The response rate for the national survey was 67 percent (1911 out of 2859 individuals). After excluding observations with missing data, our final sample size for analysis is 1789.

The survey sample captured a broad cross-section of the Lebanese population in terms of gender, age, socioeconomic status and sect (See Table 1). With this dataset, we investigate the relationship between political activism and the likelihood of individuals receiving financial aid for health services.

**Table 1:** Descriptive Statistics (N = 1789)

	**Frequency**	**% Sample**	**Receiving Health Aid**	
			**Frequency**	**%**
**Age**				
<30	268	14.98%	106	39.55%
30-40	565	31.58%	182	32.21%
40-50	632	35.33%	226	35.76%
50-60	245	13.69%	82	33.47%
>60	79	4.42%	26	32.91%
**Female**	974	54.44%	360	36.96%
**Reported household monthly ncome**				
<=500	489	27.33%	192	39.26%
501-1000	863	48.24%	281	32.56%
1001-2000	346	19.34%	113	32.66%
2001-3000	63	3.52%	24	38.10%
> = 3001	28	1.57%	12	42.86%
**Education**				
Elementary	261	14.54%	119	45.59%
Intermediate	570	31.86%	196	33.86%
Secondary	427	23.87%	128	29.98%
Vocational	131	7.32%	53	40.46%
University	352	19.68%	113	32.10%
Higher degree	48	2.68	16	33.33%
High status profession*	210	11.74%	79	37.62%
**Sectarian identity**				
Christian	782	43.71%	240	30.69%
Shia	414	23.14%	164	39.61%
Sunni	351	19.62%	137	39.03%
Druze	73	4.08%	26	35.62%
Orthodox	56	3.13%	23	41.07%
None	113	6.32%	32	28.32%
**Reported political party support**				
Free Patriotic Movement	162	9.06%	46	28.40%
Lebanese Forces	96	5.37%	34	35.42%
Kataeb	23	1.29%	3	13.04%
Future Movement	140	7.83%	74	52.86%
Hezbollah	121	6.76%	41	33.88%
Amal Movement	48	2.68%	36	75.00%
Progressive Socialist Party	44	2.46%	15	34.09%
Others	143	7.99%	61	42.66%
Do not support any party	1012	56.57%	312	30.83%

*High status professions include legislator, top manager, director, specialized professional (i.e. law, medicine), technician or assistant in a specialized profession, armed forces, and member of religious clergy.

The dependent variable, whether the individual received financial aid for health services, is a dummy variable where 1 indicates receipt of such aid and 0 otherwise. About one-third of the sample (34%) reported receiving some form of financial assistance for health care. The probability of receiving aid is slightly higher among females and surprisingly, given the purpose of the aid, highest among individuals in the highest income quintile (Table 1).

Individual political activism is measured by the Political Activity Index (PAI), which was constructed through a factor analysis of responses to eight different questions about forms of political participation, including volunteering for a political organization, attending political meetings, working for a party during elections, voting behavior, party membership, support for a party, and displaying political materials at home. The Cronbach’s alpha coefficient, an estimate for internal consistency, for PAI is 0·71. In our sample population, PAI ranges from 0 to 3·74, with a mean of 0·81 and standard deviation of 0·91.

The analyses also include variables to control for age, sex, religious commitment and piety, and a range of socioeconomic indicators, including reported household income (*income*), highest level of education attained *(education)*, employment in a profession with high social status *(high status profession)*, assets (*own*), and financial need (*need*), each of which might independently affect access to welfare. Age, reported income, education and high status profession are all categorical variables as recorded in the survey. High status professions in this study include the following occupational categories: legislator, top manager, director, specialized professional (e.g., law, medicine), technician or assistant in a specialized profession, member of the armed forces, and member of religious clergy. The own variable is an index constructed from responses to a series of questions asking whether the interviewee’s household owns a television, satellite dish, telephone, cellphone, stereo, refrigerator, car, or moped. The need variable is an index constructed from responses to questions asking whether the interviewee or his or her immediate family had problems paying rent or mortgage payments, lost a job, postponed medical treatment for financial reasons, had to borrow money, or sold personal items to raise money in the past year.

Given the dichotomous construction of the dependent variable, we employed logistic regression analysis. Standard errors are clustered at the neighborhood or village level to take into account the possible correlation of responses from households in the same geographic areas.

## Results

Table 2 presents the summary results of the logistic regression. The unadjusted odds ratio (OR) of the impact of political activism on the likelihood of receiving health aid is 1.41. Controlling for age, sex, socioeconomic status, religious commitment, and piety, political activism still (as measured by the PAI) has a significantly positive association with the probability of receiving health aid (*p <* ·*001*), with an OR of 1·44 with one unit increase in PAI. The magnitude of the impact translates into an odds ratio of 4·0 when comparing individuals with the highest political activity to those least active. On average, with all other factors held at average levels, the most politically active individuals are over two times more likely to receive financial assistance for health services than the least active ones. For example, for a woman in her forties, an increase of political activity from a PAI level of 0 to the maximum level in our sample would increase the probability of receiving financial assistance for health care from 31 percent to 58 percent. We also conducted a series of analyses to examine whether there is any interaction effect between political activism and age, sex, socioeconomic status, religious commitment, or piety, one at a time. None of the interaction was statistically significant at 95% confidence.

**Table 2:** Logistic Regression of Political Activism and Access to Financial Assistance for Health

	**Odds of receiving health aid**	
	**Unadjusted OR**	**Adjusted OR^§^**
Overall political activism (PAI)	1.41**	1.44**
Individual components of political activism		
Volunteer for Party	2.62**	2.75**
Meeting freq.	1.03	1.01
Volunteer in 05’ election	1.94**	1.98**
Vote in 05’ election	1.10	1.20
Vote list	1.00	1.02
Party member	1.78**	1.91**
Party supporter	1.49**	1.44**
Poster display	1.94**	1.87**
Age		0.94
Sex (Female)		1.36*
Income		1.10
Education		0.93
High status profession		1.29
Own		0.70**
Need		1.18
Religious commitment		1.13
Piety		0.92

^§^Adjusted for age, sex, socioeconomic status (income, education, profession), religious commitment and piety.

** p < 0.01, * p < 0.05.

Full results available at [AUTHOR WEBSITE].

**Figure 1:**
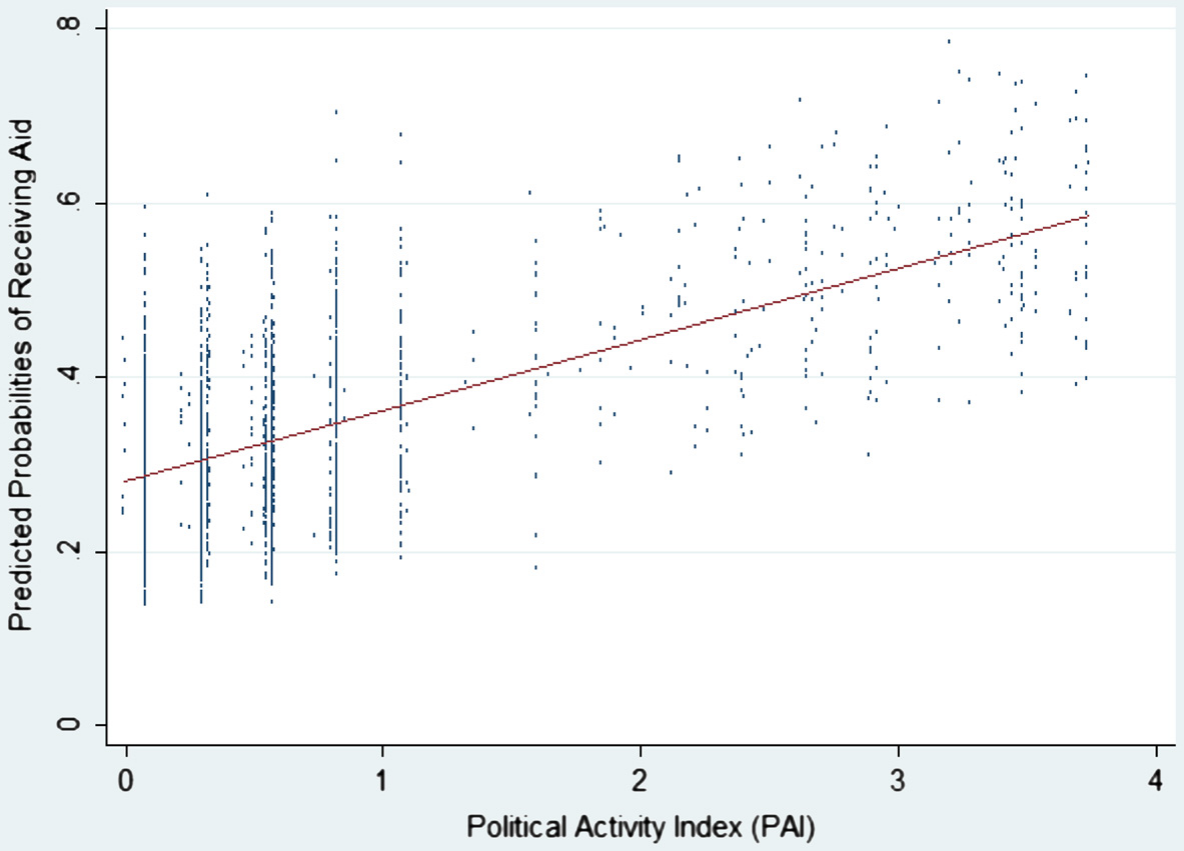
Predicted Probability of Receiving Health Aid across Levels of Political Activism.

Figure 1 illustrates the linear relationship between predicted financial access to health care and political activity among the sample population. The vertical axis of Figure 1 is the predicted probability of receiving health aid of individuals in our sample population based on the logistic regression model, while the horizontal axis depicts the value of the PAI of the same individual. The figure shows that even though there is some variation in the probability of receiving health aid for individuals of the same level of political activity, as illustrated by the vertical spread of the observation at any given PAI, on average, there is a clear positive correlation between political activism and how likely individuals could receive financial assistance to health care.

We also examine the relationship between the likelihood of receiving health aid with individual questions of political activism in order to identify whether there are specific components of political activism that have the highest correlation with health care assistance. Unadjusted and adjusted odds ratios associated with the disaggregate measures of political activism as derived from the logistic regression analyses are also presented in Table 2. The results show the relationship at the aggregate level is driven by activities indicating a higher commitment of the individuals to the parties, such as volunteering or being a party member, rather than those such as simply casting a ballot in an election or voting for a full slate of party candidates. In other words, higher investment activities have higher returns.

The empirical quantitative analyses indicate that political activism is strongly associated with the receipt of health services in Lebanon, even after controlling for a range of demographic and other socially relevant factors. Respondents in this nationally representative survey were more likely to receive financial assistance for health care if they engaged in various forms of political participation such as declaring their support for a political party, voting, shuttling others to the polls on election days, volunteering for a party and attending political meetings. As political activism increases, access to assistance for health care rises.

The logistic regression results for the socioeconomic indicators also exhibit some intriguing findings. The probability of receiving financial aid for health is significantly related to self-reported ownership of a range of household items such as household appliances, cars and mobile telephones (*own*), but not self-reported income. This is most likely due to measurement error from reporting household income and is consistent with findings that assets rather than income offer a better indication of economic status in developing countries [28] The level of education attained and employment in a high status profession also do not have a statistically significant impact on the probability of receiving financial aid.

These findings are consistent with observations derived from qualitative data, including in-depth interviews with government officials, providers and ordinary citizens as well as visits to health institutions. In Lebanon, the provision of health care and other social services can be part of a political strategy [29]. As the director of a major Shi’i Muslim charity noted, “The provision of social welfare services is both a tool and methodology that political and religious forces use to enlarge their popularity.” Similarly, in discussing the motivations for the establishment of health clinics by local NGOs, a high-level official in the Ministry of Public Health contended, “Low income areas are more apt to get new centers . . . But [NGOs] don’t do this for purely humanitarian reasons. It is either religiously or politically motivated to boost their popularity among the population.”

The qualitative interviews also revealed that the discretionary allocation of assistance for health care occurs through multiple channels. Some political organizations and religious institutions operate their own health centers, enabling them to offer subsidized or free services to supporters. These organizations also have connections to other private providers and to state officials who control access to public entitlements, allowing them to serve as mediators to third-party services and to act as gatekeepers to benefits that are supposed to be social rights available to all citizens with demonstrated need.

The discretionary allocation of health assistance according to levels of political activism by definition contributes to health inequities. Citizens with connections to politicians and parties enjoy enhanced access to health care. The director of a major hospital admitted that political leaders sometimes call him to arrange hospital beds or special treatment for their supporters: “Unfortunately, people receive financial aid not according to need but because of the requests of political leaders. The leaders of different factions . . . say ‘he’s a poor man’ and therefore ask the hospital to help one of their supporters.” Indeed, this discriminatory system may shape the channels people use to seek health assistance. Noting that some citizens do not seek social assistance from political organizations that they have not actively supported, a resident of the predominantly Shi’i southern suburbs of Beirut stated, “People do not ask for services from Hezbollah if they are not supporters because they know that they will not get much help.” Given that the largest political parties are associated with particular religious communities, partisan discrimination in the allocation of health aid can take on sectarian overtones [24,30].

## Discussions and conclusions

Our empirical quantitative and qualitative analyses have consistently shown that individuals with higher political activism have better access to health services than others because of their likelihood of receiving financial assistance. Questions may be raised that the direction of effect could be the other way around; that is, political activism is the result of receiving financial aid. Our findings about the variable impact of the disaggregated measures of political engagement provide support to our hypothesis that political activism affects access to assistance for health care, and not the reverse. For instance, our analysis suggested that having volunteered for a party in the 2005 national elections significantly increases the likelihood of financial aid for health services, although it is theoretically possible that individuals would work for a party out of gratitude for prior financial assistance. Nonetheless, because the survey was conducted in 2008, it is less likely that one would volunteer in 2005 in the expectation of the need for aid at a much later time.

The finding that higher political activism results in better financial access to health services could be driven by other factors correlated with political activism rather than the level of political activism itself, especially when individuals have higher social status whether due to income, education or occupational category. In this study, we address this potential confound by controlling for several variables that capture social status, including income, assets, education and high status profession. On average, none of these variables except assets has a statistically significant effect on the likelihood of receiving financial assistance in our results. Additionally, the findings from qualitative interviews also give us confidence that political activism itself is a significant and important determinant of financial assistance for health care in Lebanon. Future research could try to address this issue by employing instrumental variables to isolate the causal impact of political activism on health, if a viable instrument is identifiable.

This paper contributes to the growing literature on the political determinants of health and access to health care by pointing to a micro-level mechanism that shapes these outcomes, notably differential access to health financing based on levels of political participation. While most studies in this nascent literature point to formal, macro-political institutions, our analyses highlight the role of informal institutions and individual or household-level exchanges in mediating access to health care and point to a gap between de jure and de facto social rights: Although needy citizens are eligible for public coverage of treatment for certain diseases and for hospitalization, in practice their access to these “entitlements” is mediated at least in part by politicians and political organizations which exert influence over relevant agencies in Lebanon. Given that access to health care can shape health, these findings call for further research to explore whether and in what ways informal politics affect health outcomes.

Furthermore, this political dimension of health inequities exacerbates existing socioeconomic health inequalities in developing countries like Lebanon [31]. Currently in Lebanon, at most about 50 percent of the population has some kind of health insurance, generally through formal sector and government employment, although many are underinsured and/or do not enjoy de facto access to public entitlements [27]. The poor, who constitute the vast majority of the uninsured, are the most likely to turn to providers linked to political organizations for care, because, as described earlier, NGO-run health clinics and dispensaries, many run by political parties, are critical for lower-income families in urban areas and may be the only facilities available in rural areas. This makes them the most susceptible to the discretionary allocation of services by political intermediaries and they may not gain financial access despite their need. Meanwhile, ironically, the higher-income on the other hand may receive financial benefits for health care through their political connections even though they could actually afford the services by themselves.. Thus, political and socioeconomic factors may interact to further intensify health inequities. The qualitative and quantitative evidence for this phenomenon are derived from field research in Lebanon, but there is reason to believe that political behavior affects access to health care elsewhere in the Middle East and in other countries in the Global South with widely divergent levels of macroeconomic development. For example, the Sadrist Movement in Iraq and Hamas in Palestine operate their own welfare networks or mediate access to social services provided by public agencies or other non-state providers. Evidence suggests that, at various historical moments, these groups have funneled disproportionate benefits to their supporters [32,33]. In India, the Hindu Nationalist Bharatiya Janata Party (BJP) has an affiliated social welfare network that has disproportionately favored supporters, although electoral considerations have compelled it to serve beyond its base [34]. In Mexico, drug cartels run social service networks in areas where the state does not provide, funneling benefits to citizens in the areas they control [35]. Prior to the enactment of universal health coverage in 1995, supporters of the Kuomintang Party (KMT) in Taiwan received favorable access to health benefits, among other advantages. These discriminatory practices even took on quasi-ethnic dimensions as the main supporters of the KMT were predominantly from Mainland China rather than longstanding natives of the island [36].

Although each of these countries is characterized by distinct social and political cleavages, the basic principle of differential access to health care as a result of political loyalties and behavior holds true. Discriminatory access to medical care on the basis of political participation at the individual and household levels can introduce new health inequities, compounding existing imbalances along socioeconomic lines. Political institutions – and, particularly, informal institutions – are notoriously difficult to change, but enacting and enforcing policies to provide more truly universal access to health benefits are critical to ensure more equitable access to health in contexts where politics constitute a source of health disparities. Moreover, research should also carefully consider micro-level political factors in assessing equity in access to health care and health outcomes in order to inform more effective policy-making.

## Competing interests

The authors declare that they have no competing interests.

## Authors’ Contribution

MC collected the data. BC undertook the empirical analysis. Both authors contributed equally to the interpretation of the findings and drafting of the report.
